# Emerging Nonvolatile Memory Technologies in the Future of Microelectronics

**DOI:** 10.1021/acsomega.5c01414

**Published:** 2025-06-30

**Authors:** Linda Katehi, Su-in Yi, Yuxuan Cosmi Lin, Sarbajit Banerjee, Qiangfei Xia, J. Joshua Yang

**Affiliations:** 1 Electrical Engineering and Materials Science and Engineering 14736 Texas A&M University, College Station, Texas 77843, United States; 2 Chemistry Department, Texas A&M University, College Station, Texas 77843, United States; 3 Electrical and Computer Engineering, 14707 University of Massachusetts, Amherst, Massachusetts 01003, United States; 4 Electrical Engineering and Computer Science University of Southern California, Los Angeles, California 90007, United States

## Abstract

Memory technologies are central to modern computing systems, performing essential functions that range from primary data storage to advanced tasks, such as in-memory computing for artificial intelligence (AI) and machine learning (ML) applications. Initially developed solely for data retention, these technologies are evolving to support new paradigms, such as in-memory computing, where processing occurs directly within the memory array. This evolution significantly enhances computational efficiency by minimizing data transfer between processors and memory, resulting in increased speed and reduced energy consumption, critical factors for AI and ML workloads. Such demanding requirements are driving innovations beyond traditional complementary metal-oxide semiconductor (CMOS) technologies. Emerging nonvolatile memories (eNVMs) represent a promising class of technologies designed to replace or augment conventional volatile memories, such as random-access memory (RAM). Unlike RAM, which loses stored information when the power is disconnected, eNVMs maintain data integrity during power interruptions and system shutdowns. This review examines a range of emerging memory materials and device architectures, including resistive random-access memories (ReRAMs), magnetic random-access memories (MRAMs), ferroelectric random-access memories (FeRAMs), and phase-change memories (PCMs). Additionally, novel eNVMs based on two-dimensional (2D) and organic materials are explored, along with a discussion of the transition from digital to synaptic computing and the potential it offers to address significant technological barriers that may impede the use of AI in accelerating discovery. The discussion encompasses a comprehensive analysis of technological advancements, current development trajectories, and the challenges that still need to be addressed.

## Graphical abstract

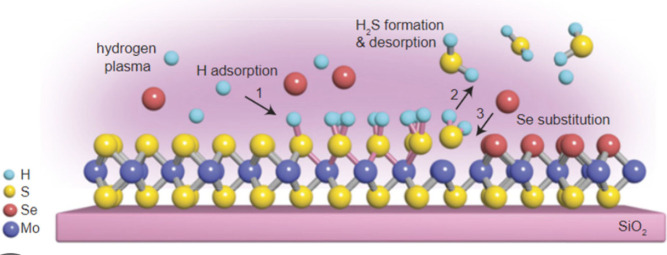

## The Role of Memories in the beyond-CMOS Era

A widely stated challenge in developing beyond-CMOS microelectronics is finding an alternative to von Neumann’s computing and processing (Figure 1). There is an acute need for new memory devices that combine the best features of today, including compatibility with CMOS process flow and the ability to scale beyond the current limits of SRAM (static random access memory) and flash memory. A memory technology with these attributes would be suitable for both standalone and embedded memory applications in analog and digital processing. According to the 2022 International Roadmap for Devices and Systems (IRDS),^1^ such technology is expected to initiate a revolution in computer architecture. Research on nonvolatile memories began with the development of charge memory devices in the 1960s. It continued for another three decades until 2010, when embedded semiconductor memories reached 28 nm,^2,3^ and encountered a barrier in size reduction due to charge leakage. Nonvolatile memory offers essential advantages, and the degree of nonvolatility is measured by the time that data can be retained. Flash memory is considered the baseline nonvolatile memory because it is highly mature, well-optimized, and has a significant commercial presence.

**Figure 1:**
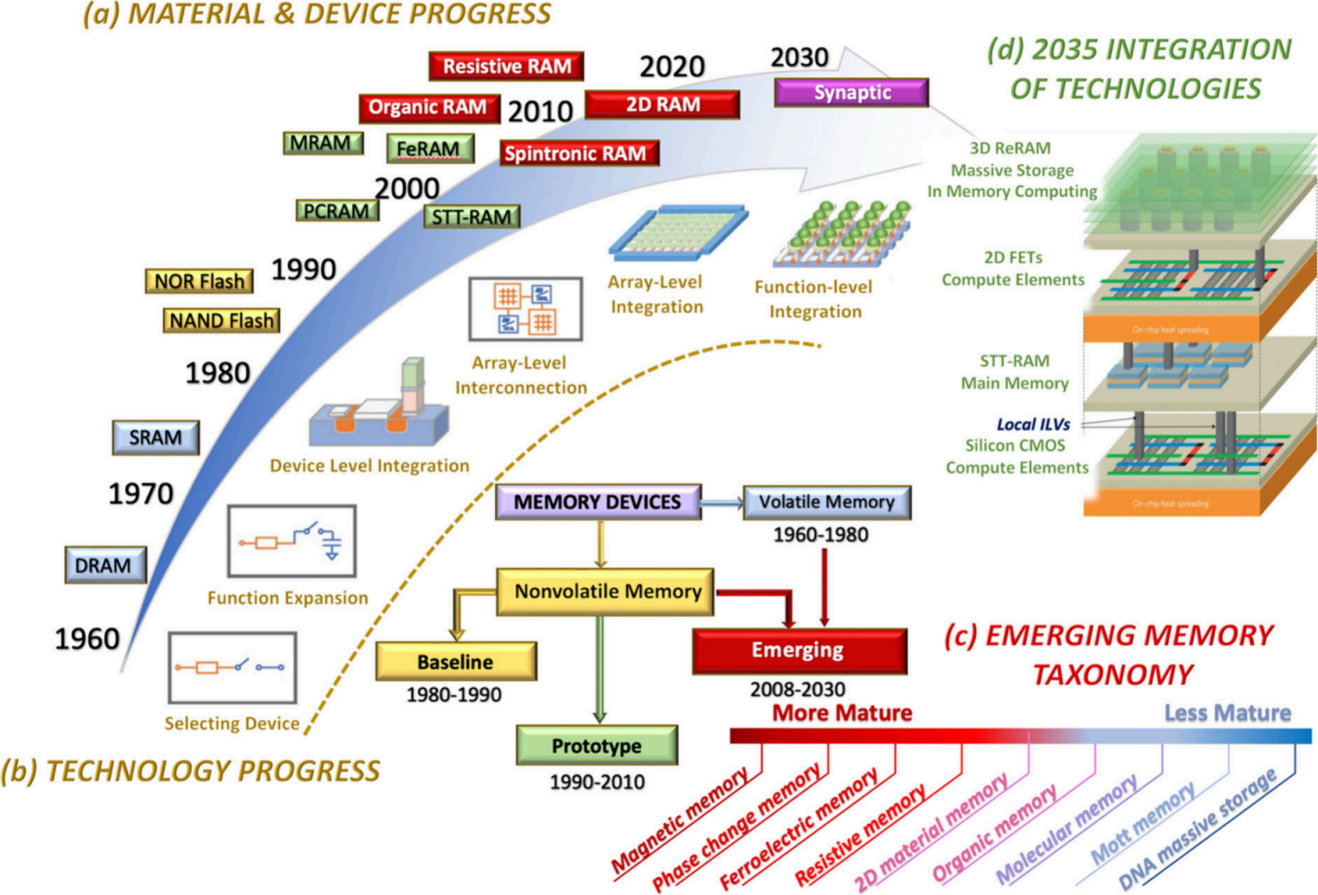
(a) A simple visual method showing the history of developing nonvolatile memories. (b) Timeline of progress. (c) Taxonomy of Emerging Memory Devices categorized by their maturity level. (d) The 2035 projected circuit architecture is characterized by integrating various emerging memory technologies selected to meet the requirements of the design chips’ functions.

Prototypical memory technologies have matured to a point where they are commercially available and possess a comprehensive scientific, technological, and systems knowledge base in the literature. The inability of charge-memory devices to achieve nanometer-layer thickness has redirected technological interest toward the three-dimensional stacking of NAND flash cells and to “emerging” memory devices^4–6^ . Figure 1c identifies a pipeline of six major classes of emerging memories ranging from more mature to less mature: Novel Magnetic Memories (MRAM), Ferroelectric Memories (FeRAM),^7^ and Oxide-Based Resistive Memories (ReRAM)^8–10^ have demonstrated appealing properties and are ready for demonstration to pave the way for commercialization. Phase Change Memories (PCM or PRAM),^18^ Conductive Bridging Memories (CBRAM), 2D Material Memories (2D RAM), and Organic and Molecular Memories^11–13,21^ are less mature but still ripe for technological innovations^10,13–15^ . Other Molecular Memories, such as Mott memories and DNA-based data storage, remain in the early stages of discovery^16,17^ . Memory-based computing and processing will become indispensable to future computing systems, thanks to the unique characteristics of emerging memories, including nonvolatility, byte addressability, high density, high scalability, and the need for near-zero standby power. The integration of Emerging Memories with Synaptic Memories,^23–26^ which are expected to flourish in the next few years, is anticipated to revolutionize future computing architectures and enhance existing systems in terms of performance, energy efficiency, and processing capabilities, extending from storage systems to edge and cloud environments, as well as database systems and blockchain decentralized applications.^2,19,20^

## Memory Diversity and Its Advantages

The diversity of emerging memory technologies, such as Ferroelectric Memories (FeRAM), Redox Resistive Memories (ReRAM), Magnetic Memories (MRAM), Phase Change Memories (PCM), and Organic and Molecular Memories (OMRAM), caters to specific application needs by offering designers a variety of options based on their desired specifications and operating environments. Each technology offers a distinct set of advantages, including durability, efficiency, and suitability for specific environments or tasks.

Research into high-temperature, nonvolatile memory addresses the need for memory systems to operate reliably under extreme conditions. This research fills a significant gap in the technology market and expands the potential applications of memory devices in harsh environments. Innovations in material selection and manufacturing precision enable memory devices to operate reliably under extreme conditions, including high temperatures and high radiation, without degradation—a crucial attribute for the aerospace and geothermal exploration industries.

In the rapidly evolving field of memory technologies, two-dimensional (2D) materials have emerged as a promising frontier due to their unique physical properties and scalability. These materials possess the potential to revolutionize memory devices because of their atomic-scale designability and compatibility with existing technologies. Positioned to transform the memory device market, two-dimensional (2D) materials showcase distinctive attributes including atomic-scale thickness and design flexibility. They facilitate the creation of memory devices that are not only faster and more energy-efficient but also capable of seamlessly integrating with current technologies, thereby enhancing the overall performance of electronic systems. The scalability of 2D materials paves the way for mass production of memory devices. With ongoing advancements in synthesis and transfer processes, these materials are becoming increasingly accessible for broader applications, heralding a new era of memory technology development that could meet the future demands of computing and data storage.

Unlike conventional memory technologies, ReRAM and Synaptic RAM support in-memory computation, offering nonvolatility that enables low-latency and energy-efficient data processing. Their ability to perform analog multiply and-accumulate operations directly within memory arrays eliminates the need for energy-intensive data transfers between memory and processors—a bottleneck in conventional von Neumann architectures. This makes them ideal for edge computing systems, where real-time inference, low power consumption, and compact design are critical design characteristics.

These memory technologies are particularly well-suited for neuromorphic and adaptive systems. Synaptic RAM, inspired by biological synapses, can implement learning rules such as spike-timing-dependent plasticity (STDP), enabling hardware-based learning and real-time responsiveness in dynamically changing environments. This capability is essential for autonomous IoT devices that learn from and adapt to new stimuli without continuous cloud connectivity. Furthermore, the nonvolatility of xRAM (ReRAM, FeRAM, MRAM,) or PCM memories enables IoT systems to retain operational states across power cycles, thereby enhancing reliability and allowing for instant-on functionality in power-constrained or intermittently powered environments.

As the demand for intelligent, decentralized systems grows, xRAM and Synaptic RAM provide pathways to scalable, low-power, and robust computing platforms. Their high density, compatibility with 3D integration, and potential for monolithic integration with CMOS circuits make them central to the evolution of hardware for AI and IoT. These technologies support the vision of distributed intelligence, enabling seamless, autonomous, and context-aware computing across a wide range of applications, including smart sensors, edge analytics, and beyond.

## Nonvolatile Memories on Flexible Substrates: A State-of-the-Art Perspective

The integration of nonvolatile memory (NVM) technologies on flexible substrates has gained significant momentum, driven by emerging applications in wearable electronics, soft robotics, and distributed Internet of Things (IoT) systems. These systems require memories that not only retain data without power but also withstand mechanical deformation, such as bending, stretching, and twisting. Among the various NVM technologies, ReRAM and FeRAM have demonstrated the most advanced performance on flexible platforms. ReRAM, with its simple metal–insulator–metal structure and compatibility with low-temperature processing, has shown excellent mechanical endurance and retention on polymer substrates such as PET and polyimide. FeRAM, particularly those using P­(VDF-TrFE) or doped HfO_2_, provides low-voltage switching and stable polarization states, demonstrating reliability over thousands of mechanical cycles. Although more challenging, limited demonstrations of PCM and early explorations of flexible MRAM based on organic or 2D magnetic materials are also underway.

Organic^21,22^ and molecular memory technologies have recently been investigated, showing great promise for AI edge computing and bioinspired devices. Organic materials with tunable molecular structures and their associated electrical, optical, thermal, and chemical properties can serve as alternatives to conventional memristive devices in specific neuromorphic computing algorithms.^23^ The volatile and dynamic electrical characteristics of organic materials and devices can emulate the functionalities of biological neuronal and synaptic responses, including spike-timing-dependent plasticity, spike-rate-dependent plasticity, and both long- and short-term plasticity.

Recent advances in materials and fabrication techniques—such as inkjet printing, transfer printing, and room-temperature deposition—have enabled the fabrication of nonvolatile memory (NVM) directly on plastic substrates without compromising mechanical integrity. Hybrid material systems utilizing 2D materials and nanostructured dielectrics have enhanced the device performance under strain. Despite these advances, challenges persist in achieving long-term mechanical reliability, maintaining data retention under flexure, and integrating flexible nonvolatile memory (NVM) with logic and sensing elements into fully flexible systems. Future work will concentrate on monolithic integration, additive manufacturing of entire memory arrays, and developing robust encapsulation methods for stable operation in dynamic environments.^25^

As these hurdles are overcome, flexible nonvolatile memories (NVMs) will form the backbone of adaptive, conformable electronics that seamlessly integrate with their surroundings. Flexible neuromorphic integrated circuits can be easily combined with organic and molecular memory technologies. In these applications, neural network models can be trained on a centralized, more powerful AI processing chip. After training, the model can be transferred to flexible neuromorphic integrated circuits, which can be positioned near biochemical sensors and serve as localized processing units for these distributed sensors. Both the unique functionalities and application scenarios provided by organic and molecular memory technologies further expand the scope of AI computing to distributed computing and sensing systems in wearable and implantable biomedical applications.

The critical knowledge gaps that limit the adaptation of emerging memory technologies include (a) eliminating material impurities and nonuniformities that lead to limited write endurance and a shorter lifetime, (b) identifying nonlinear dynamics and analog noise that impact memory usage in computing and processing, and (c) reducing the time required for memory reprogramming along with associated write energy consumption.

## Realization and Use of Emerging Memories

The fabrication of emerging memory materials requires ultrahigh vacuum conditions during deposition processes. Sophisticated fabrication facilities ensure that memory devices are produced with high precision and remain free from contaminants. The development and use of integrated tools for deposition and characterization facilitate the creation of memory devices with precise material properties and structural integrity. Such tools enable seamless transitions between different stages of device fabrication, ensuring that the purity and functionality of the materials remain intact throughout the process. This not only enhances memory device performance but also significantly extends their operational lifespan and reliability.

Understanding the fundamental properties of the materials used in memory devices is essential. Mistakes made early in development can lead to significant performance issues later. The use of emerging memories in storage applications requires rapid and low-energy switching, enabling data to be written and read efficiently while consuming minimal power.^27^ In contrast, memory devices must maintain stability after programming to ensure reliable in-memory computing. Once programmed, the device is read multiple times during computation, and demonstrating repeatability and endurance during these reads is crucial for accurate processing. Improvements in characterization techniques, such as in situ measurements and detailed statistical analysis across different device regions, offer a deeper understanding of material behavior and performance. This leads to more consistent and reliable memory devices, which are vital for their application in high-stakes industries. The development of multichannel testing systems for emerging memory devices facilitates more efficient and precise testing processes.^28^ These testing systems are essential for designing and developing energy-efficient hardware that can meet the computational demands of AI models, significantly reducing the energy consumption in these technologies. Each of these advancements addresses specific challenges within the memory technology sector, setting the stage for future innovations that could redefine how memory devices are integrated and utilized across various technology platforms. As these technologies evolve, they continue to push the boundaries of what is possible in computing, heralding a new era of speed, efficiency, and reliability.^29^ Memory technology development not only highlights potential innovations and technological breakthroughs but also emphasizes the present challenges that need to be tackled. These challenges can be grouped into five categories: (a) material synthesis, fabrication precision, and characterization; (b) device scalability, lifetime expendability, and reproducibility; (c) material-device multimodal characterization; (d) device interconnectivity and compatibility to existing and new CMOS technologies; and (e) packaging and heterogeneous integration.

**2:**
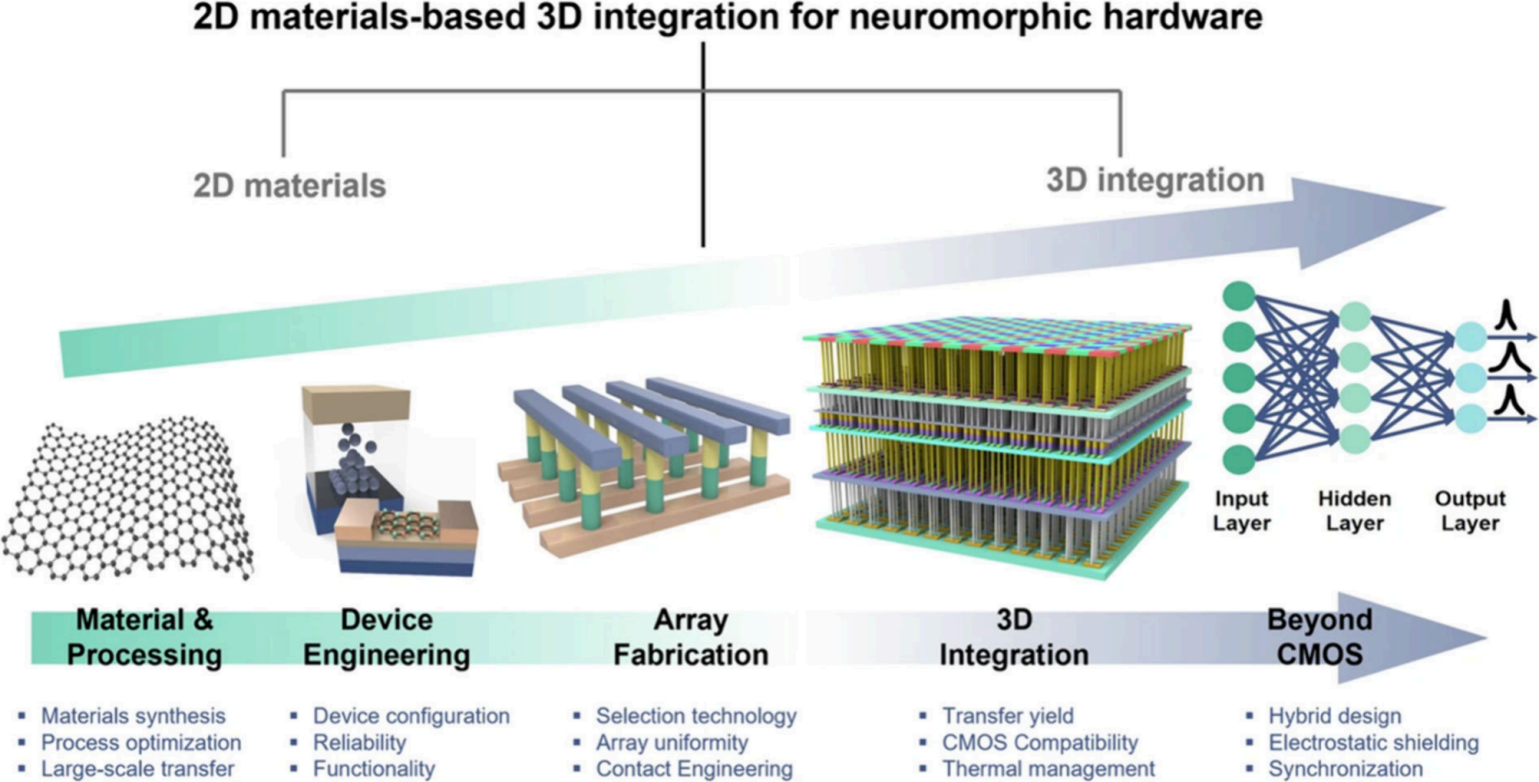
A schematic of the 3D integrated neuromorphic hardware roadmap using 2D materials. Reprinted with permission from Kim, S.J., et al, “2D materials-based 3D integration for neuromorphic hardware,” *NPJ 2D Mater. Appl.* 8, 70 (2024), Nature.

In the case of material synthesis, selecting materials that can withstand extreme conditions, such as high temperatures and densities, while meeting the computational demands of AI and ML applications is challenging. Additionally, materials must be precisely composed to ensure stability and functionality. Developing AI/ML methods for selecting new composite materials can accelerate the synthesis process. Achieving high-quality materials requires manufacturing precision, which is essential for avoiding defects that could degrade memory performance. This includes maintaining ultrahigh vacuum conditions to prevent contamination during the thin-film deposition process. High-quality materials can provide device stability, reproducibility, and scalability.^30^

Two-dimensional (2D) materials are critical to the advancement of emerging nonvolatile memory (eNVM) technologies due to their unique electrical, mechanical, and thermal properties at atomic thickness. Their atomically thin nature enables aggressive scaling beyond traditional semiconductor limits, allowing ultrahigh-density memory integration with low power consumption. Materials such as graphene, transition metal dichalcogenides (e.g., MoS_2_, WS_2_), and hexagonal boron nitride offer high carrier mobility, tunable bandgaps, and defect-engineered switching characteristics, which are ideal for resistive switching, ferroelectric behavior, and charge-trapping mechanisms in emerging nonvolatile memory (eNVM) devices. Moreover, their mechanical flexibility and chemical stability make them suitable for flexible and wearable electronics, where traditional materials fall short. These characteristics enable the realization of next-generation memory devices with fast switching speeds, endurance, and retention, which are essential for in-memory computing and AI applications. Despite the great potential of technology in memory devices, two-dimensional materials present a unique set of challenges. Consistently producing large-area, high-quality single-crystal 2D materials poses a significant technological hurdle that must be overcome to enable widespread application. These materials are susceptible to environmental factors, including oxygen and moisture, which can compromise their properties.^18,30^ Developing effective encapsulation and protection strategies is crucial for the practical use of these materials. Integrating 2D materials with existing manufacturing processes, particularly CMOS technology, requires innovations in low-temperature growth techniques and damage-free transfer methods.^31^

To meet the high standards necessary for commercial viability and fabrication precision, advanced characterization techniques are essential. Achieving reproducibility in characterization across different device sections is critical yet challenging due to variability in material behavior and defects. Real-time, in situ measurements are vital for understanding the dynamic interactions within the memory device during operation and pose significant technical challenges, especially in adapting tools like transmission electron microscopes (TEM) for live electrical biasing.^32^ The development of memory technologies capable of operating at extreme temperatures faces a significant challenge: creating materials that can withstand repeated temperature-induced stress while maintaining data integrity. Additionally, integrating these memory technologies with other high-temperature-capable components, such as silicon carbide (SiC) transistors, poses considerable engineering challenges to ensure reliability and performance.^33^ Developing energy-efficient hardware for AI or neuromorphic applications requires testing systems that can handle the high parallelism demanded by emerging memory technologies such as memristors; this is a complex and technically challenging task. Integrating memory devices with in-memory computing capabilities into AI hardware platforms necessitates overcoming significant barriers in system architecture and device interoperability.

Furthermore, developing interconnect technologies that can manage the density and scalability required for AI applications such as GPT-3 remains a formidable challenge. Advanced bonding techniques are crucial for integrating dense memristor arrays with other system components without compromising-performance. These techniques play a transformative role in the development of next-generation computing systems for artificial intelligence (AI), machine learning (ML), and Internet of Things (IoT).

## The Digital to Synaptic Transition in Future Computing Systems

Modern computing systems are built on digital architectures optimized for speed, precision, and logical determinism. While these systems excel in conventional tasks, they are increasingly strained by the demands of data-intensive artificial intelligence (AI), machine learning (ML), and Internet of Things (IoT) applications. Traditional von Neumann architectures suffer from the Memory Wall, where the energy and latency costs of data movement between memory and the processor dominate total system performance.^34^ This Memory Wall has sparked growing interest in brain-inspired computing paradigms, particularly those that leverage synaptic behavior to achieve localized and energy-efficient computation.

**3:**
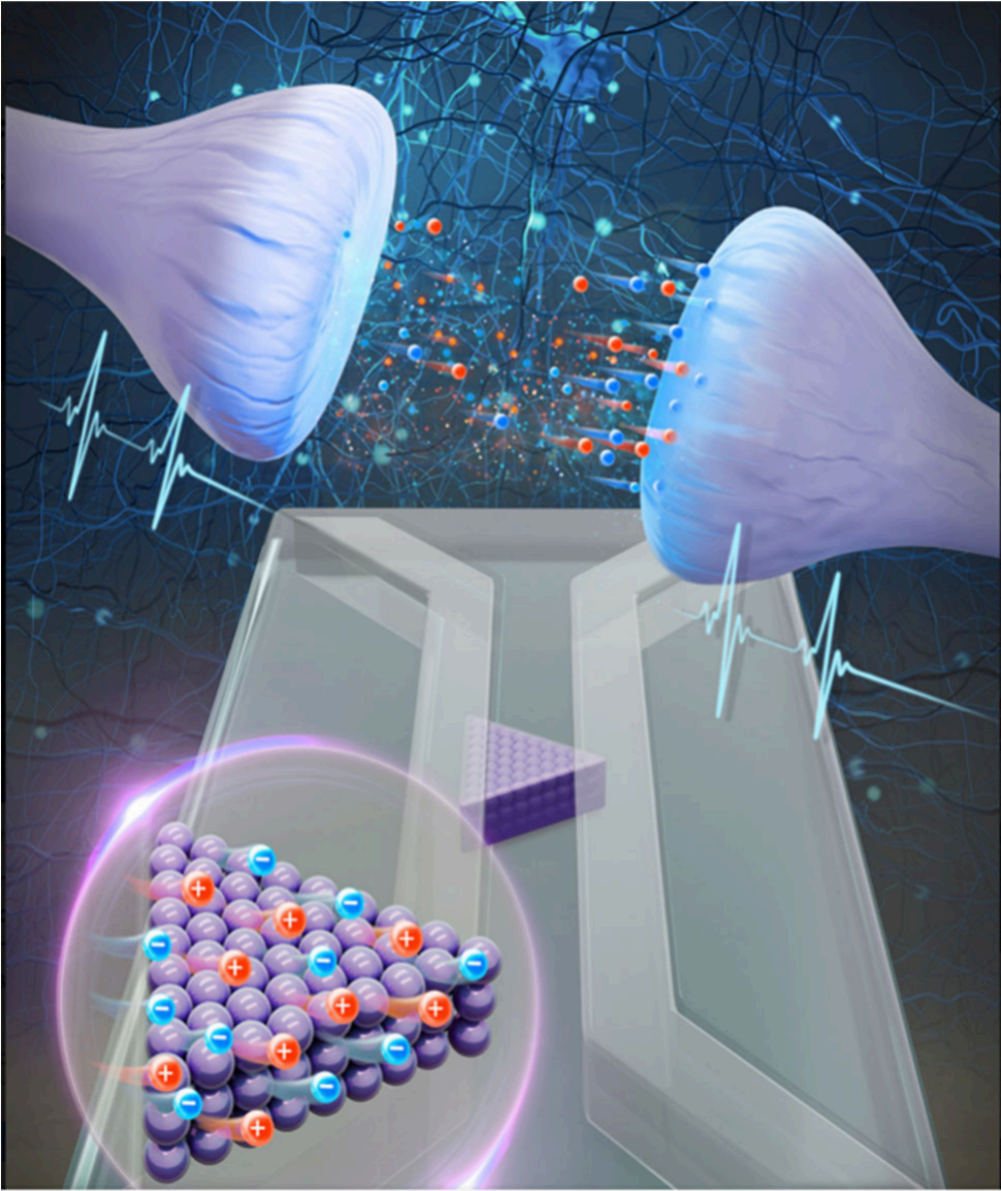
Brain-inspired computing with fluidic iontropic nanochannels. Reprinted with permission from Kamsma, T. M., *Nanochannels*, Vol. *121*, No. 18, (2024), PNAS.

A digital-to-synaptic transition refers to the progressive shift from discrete, logic-based digital operations to analog or event-driven synaptic behaviors that emulate biological computation.^35,36^ In current hybrid systems, analog and digital inputs are translated into synaptic-like signals, such as current pulses or voltage waveforms, which directly control memory states or neuronal activations in memristive or neuromorphic devices (Intel). These transitions are already manifesting in edge AI devices, neuromorphic coprocessors, and experimental crossbar arrays that combine storage and processing within a single physical layer. Such architectures enable in-memory computation and real-time learning using biologically inspired mechanisms including spike-timing-dependent plasticity (STDP). However, the transition from analog to digital and from digital to analog spikes places additional energy demands on the system, thereby reducing its entropy. In systems where information is received in analogue form, a direct transition from analogue to spikes will catalyze the development of fully synaptic systems, where processing and computing are dominated by sparse, asynchronous, and local interactions—akin to those found in the human brain. Future AI systems will increasingly employ end-to-end analogue computation, eliminating the need for centralized digital logic for many tasks. Spiking neural networks (SNNs) and event-driven architectures are poised to become mainstream, integrating with novel sensors, such as dynamic vision sensors (DVS) to form closed-loop systems that respond and adapt to real-world stimuli in real-time. These systems will offer significant improvements in energy efficiency, responsiveness, and adaptability, which are critical for autonomous agents, intelligent sensors, and wearable devices.

In systems in which information is transmitted in digital form, the digital-to-synaptic transition signifies a fundamental shift in how computation is conceived and implemented. Rather than serving as the core computational engine, digital logic functions as an interface to manage and orchestrate synaptic processes. As advancements in materials, devices, and algorithms converge, synaptic computing will enable the next generation of intelligent, distributed, and adaptive systems, expanding the capabilities of AI, ML, and IoT beyond current limitations.

## Summary

In summary, the technological opportunities, materials, and integration challenges presented in this article reflect the complexity of advancing memory technology across various domains. Each domain requires targeted research and development efforts to overcome specific technical, environmental, and operational hurdles. Addressing these challenges is essential for advancing memory technologies, which are crucial for future computing, AI, and advanced sensing and imaging applications. Providing appropriate instrumentation to a rapidly growing community of researchers will accelerate technological advancements and innovations with significant implications for the development of new materials, devices, products, and market offerings. This will impact the full scale of a nation’s economic growth and establish the US as a leader in the emerging technology field. In the 1970s, 1980s, and 1990s, a boom occurred in US-based microelectronics facilities, primarily concentrated in and around major industry research centers and a select group of academic institutions. Over the past 25 years, the opening of international markets and globalization of the microelectronics industry have spurred the manufacturing and design of tools for industries worldwide, offering commercial products and technology solutions across the globe. This expansion of capabilities and products also underscores an urgent need to reduce energy consumption and limit the exponential growth of data, while addressing the demand for new technology solutions. We must either extend our capabilities beyond CMOS or create new technological innovations. This will require new materials, devices, and memory technologies. It will necessitate tools specifically designed for the fabrication and metrology of emerging materials as well as making these tools accessible to the broader research community. We need to democratize research tools, data, and knowledge, empowering the research community to accelerate innovation in this area. We can achieve this goal only by creating a national resource for expertise, technological innovation, and workforce development that supports research, training, and education across all engaged communities.
